# Anatomy of the greater palatine foramen and canal and their clinical significance in relation to the greater palatine artery: a systematic review and meta-analysis

**DOI:** 10.1007/s00276-022-03061-z

**Published:** 2023-01-14

**Authors:** Dong Woon Kim, Jonasz Tempski, Jan Surma, Jakub Ratusznik, Wiktor Raputa, Izabella Świerczek, Jakub R. Pękala, Iwona M. Tomaszewska

**Affiliations:** 1 International Evidence-Based Anatomy Working Group, Department of Anatomy, Jagiellonian University Medical College, Kraków, Poland; 2 Center for Innovative Medical Education, Department of Medical Education, Jagiellonian University Medical College, Kraków, Poland

**Keywords:** Greater palatine artery, Greater palatine foramen, Greater palatine canal, Hard palate, Meta-analysis, Systematic review

## Abstract

**Purpose:**

Accurate knowledge of greater palatine foramen (GPF) and greater palatine canal (GPC) anatomy is necessary to avoid injury to the greater palatine artery (GPA) when performing a variety of anesthesiologic, dental or surgical procedures. The aim of this paper was to perform a systematic review and meta-analysis of literature on the anatomy and localization of bony structures associated with the GPA, namely the GPF and GPC.

**Methods:**

A systematic literature search was performed using PubMed, Embase, ScienceDirect, and Web of Science databases. Seventy-five studies were included in the meta-analysis (*n* = 22,202 subjects).

**Results:**

The meta-analysis showed that the GPF is positioned 17.21 mm (95% CI = 16.34–18.09 mm) from the posterior nasal spine, 2.56 mm (95% CI = 1.90–3.22 mm) from the posterior border of the hard palate, 46.24 mm (95% CI = 44.30–48.18 mm) from the anterior nasal spine, 15.22 mm (95% CI = 15.00–15.43 mm) from the midline maxillary suture, 37.32 mm (95% CI = 36.19–38.45 mm) from the incisive foramen, and opposite the third maxillary molar (M3) in 64.9% (58.7–70.7%) of the total population.

**Conclusion:**

An up-to-date, comprehensive analysis of GPF and GPC clinical anatomy is presented. The results from this evidence-based anatomical study provides a unified set of data to aid clinicians in their practice.

## Introduction

The hard palate is formed by the fusion of the palatine processes of the maxilla and the horizontal plates of the palatine bone at the so-called transverse palatine suture [20].

The mucosa of the hard palate is predominantly supplied by the greater palatine artery (GPA), which originates from the descending palatine artery in the pterygopalatine fossa, descends through the greater palatine canal (GPC), and emerges from the greater palatine foramen (GPF) near the posterior border of the hard palate [46, 51, 52]. The location of the GPF varies, but it can generally be identified by palpation of the palate opposite the third maxillary molar teeth [35, 60, 74]. Viveka et al. [83] concluded that the utilization of multiple anatomical reference points, such as the incisive foramen, the midline maxillary suture, and the second and third maxillary molars, simplifies identification of the GPF. Adequate identification of the GPF allows for visualization of arterial pulsations, and confirms the location of the GPA.

At the hard palate, the GPA courses anteriorly in close proximity to the alveolar ridge. The greater palatine nerve traverses a groove medial to the artery, from which it is separated by a palpable crest, which can be used by clinicians to localize both structures [13, 60]. The main trunk of the GPA—the lateral branch—enters the nasal cavity through the incisive foramen [50, 60], where it anastomoses with the posterior septal branch of the sphenopalatine artery to supply the anteroinferior portion of the nasal septum. The diameter of the GPA is greatest at the site of its emergence from the GPF, and then decreases gradually as it courses toward the incisive foramen. The GPA gives off most of its branches in the premolar area, and more commonly toward the alveolar side, rather than to the hard palate [28].

An accurate appreciation of the GPA’s location and size is essential to avoid its injury and the resulting surgical and post-surgical complications [69]. Bleeding from the GPA can be difficult to control, with the potential to cause significant blood loss and palatal tissue necrosis [16]. The injury itself, or damage caused by attempts to arrest hemorrhage, may lead to postoperative pseudoaneurysms, or injury to the greater palatine nerve, resulting in paresthesia or insufficient anesthesia of the ipsilateral hard palate [17], and in rare cases, transient ophthalmoplegia [21, 22].

Injury to the GPA occurs most commonly during subepithelial connective tissue graft harvesting and can result in prolonged intraoperative bleeding and postoperative wound healing complications related to impaired blood flow [14, 72]. In fact, the position of the GPA, along with the thickness of the palatal mucosa, are the two main factors that dictate the size of subepithelial connective tissue grafts that can be safely harvested from the hard palate [16].

GPA injury may also be implicated during down-fracture of the maxilla [12], or in other surgical procedures such as osteotomy of the medial and lateral maxillary sinus walls, pterygomaxillary disjunction, endoscopic medial maxillectomy [13], and pterygopalatine fossa infiltration [12]. The last procedure involves injecting either a vasoconstricting agent into the greater palatine canal––to prophylactically induce hemostasis and limit posterior epistaxis during endoscopic sinus surgery and septorhinoplasty––or an anesthetic solution through the greater palatine canal into the pterygopalatine fossa, to achieve anesthesia of the hemi-maxilla during dental procedures by maxillary nerve block [11]. Clinicians can increase the efficiency and safety of these procedures by referring to the anatomical structures in the oral cavity when determining the adequate position, angle, and length of the needle used for pterygopalatine fossa infiltration [16].

Lastly, the morphological parameters discussed are of clinical significance in the mobilization of GPA for closure of oroantral fistula using mucoperiosteal pedicled palatal flaps [16]; radical release of the GPA during cleft palate repair and reconstruction [26]; and endoscopic cauterization of the GPA at the incisive foramen for the purpose of controlling recurrent or uncontrolled anterior epistaxis [15].

We aimed to update and extend the methodology outlined by Tomaszewska to conduct the meta-analysis on the location of the GPF relative to the maxillary molars, by applying it to other anatomical data extracted from the studies. The objective of our review was to update and extend that of Tomaszewska et al. [75] in 2014. The protocol was methodologically planned and followed, although it was not registered. An updated search strategy was utilized to broaden the scope of the research question to include all available anatomical data to synthesize as evidence by introducing more keyword phrases that describe other related anatomical structures than the GPF.

The main objective was to synthesize evidence from all available studies reporting anatomical data, including cadaveric (i.e., dry skulls) and CT-imaging studies of adult patients (i.e., ≥ 21 years old), combining the results into a comprehensive set of readily available data. The primary outcomes to be measured were the pooled mean estimates of the distances between the center of the GPF and five major anatomical reference points, GPF and GPC diameters, and length and angle of the GPC; and the pooled prevalence estimates of the location of the GPF relative to the maxillary molar teeth, morphology of the GPF, and direction of GPF opening into the oral cavity. Secondary outcome measures included subgroup analysis based on the geographical region of the studies included in the analysis, to probe for sources of heterogeneity.

## Materials and methods

### Search strategy

The authors strictly followed the PRISMA (Preferred Reporting Items for Systematic Reviews and Meta-Analyses) [57] guidelines throughout the literature search [Online Supplementary File 1].

The major electronic databases (PubMed, Embase, ScienceDirect, and Web of Science) were searched extensively to identify articles eligible for inclusion in our meta-analysis up to July 2022. No lower date limit was applied. The following search terms: “greater palatine artery”, “greater palatine canal”, “greater palatine foramen”, “pterygopalatine fossa anatomy”, “pterygopalatine canal”, and “descending palatine canal” were used in different combinations, as shown in Table 1. The references in all included articles were searched manually to identify any further relevant publications. We included only published studies, relying on the journal review process as one step of quality control.

**Table 1:** Full database search strategies

Database	Full search strategy
PubMed	(greater palatine artery) OR (greater palatine canal) OR (greater palatine foramen) OR (pterygopalatine fossa anatomy) OR (pterygopalatine canal) OR (descending palatine canal)
EMBASE	(greater AND palatine AND artery) OR (greater AND palatine AND canal) OR (greater AND palatine AND foramen) OR (pterygopalatine AND fossa AND anatomy) OR (pterygopalatine AND canal) OR (descending AND palatine AND canal)
ScienceDirect	(“greater palatine artery”) OR (“greater palatine canal”) OR (“greater palatine foramen”) OR (“pterygopalatine fossa anatomy”) OR (“pterygopalatine canal”) OR (“descending palatine canal”)
Web of Science Core Collection/SciELO/BIOSIS/Current Content Connect/Korean Journal Database/Russian Citation Index	(((((ALL = (greater palatine artery)) OR ALL = (greater palatine canal)) OR ALL = (greater palatine foramen)) OR ALL = (pterygopalatine fossa anatomy)) OR ALL = (pterygopalatine canal)) OR ALL = (descending palatine canal)

Full search strategies used to search major electronic databases. Databases were accessed on July 2022

### Eligibility

Study eligibility for inclusion in our meta-analysis was assessed independently by two reviewers (J.R. and W.R.). Studies were considered eligible for inclusion if they (1) were cadaveric or imaging studies, and (2) reported relevant and extractable data on the clinical anatomy of the greater palatine artery, foramen, or canal. The reviewers did not consider (1) case reports, systematic reviews, animal studies, letters to editors, or meta-analyses, (2) studies that provided missing, unclear, or incomplete results, and (3) studies that did not clearly define (by text or figures) the descriptive anatomy used in the study [33]. Review of full-text articles was limited to the ones published in English language. All differences of opinion among the reviewers concerning the eligibility of the studies were resolved by consensus through consultation with a third reviewer (D.K.).

### Data extraction

The studies were analyzed looking for all numerical parameters that could be directly compared between studies. This meant that the same parameter was used in at least two different studies and measured with a comparable degree of precision. The following parameters were included:

1. Distance between the GPF and the posterior nasal spine (GPF–PNS)
2. Distance between the GPF and the posterior border of hard palate (GPF–PBHP)
3. Distance between the GPF and the anterior nasal spine (GPF–ANS)
4. Distance between the GPF and the midline maxillary suture (GPF–MMS)
5. Distance between the GPF and the incisive foramen (GPF–IF)
6. Location of the GPF in relation to the second (M2) and third (M3) maxillary molars
7. Diameter of the GPF in anteroposterior (AP) and lateromedial (LM) dimensions
8. Shape of the GPF
9. Direction of GPF opening into the oral cavity
10. Angle of the GPC relative to the vertical plane and to the transverse plane
11. Length of the GPC
12. Diameter of the GPC upper opening in the anteroposterior (AP) dimension

### Quality assessment

The authors used the AQUA tool to evaluate both the quality and accuracy of the anatomical studies incorporated into this meta-analysis, as well as to properly classify their quality and risk of biases [32]. The assessment covers five domains: (1) objective(s) and study characteristics, (2) study design, (3) methodology characterization, (4) descriptive anatomy, and (5) reporting of results. The potential risk for bias in each domain is appraised by judging it as “low,” “high,” or “unclear” using the signaling questions with answers “yes,” “no,” or “unclear,” respectively. In other words, all queries answered with “yes” place the corresponding domain in the “low” risk of bias category, whereas all queries answered with “no” place the corresponding domain in the “high” risk of bias category. Inadequate data that did not allow for clear scrutiny were placed in the “unclear” risk of bias category.

### Statistical analysis

The extracted data were pooled into a meta-analysis using R software, with the ‘meta’ package (R Foundation for Statistical Computing). The inverse-variance, random-effects model was used to calculate the pooled effect size estimate across the studies, and the DerSimonian–Laird method was used to estimate the between-study variance, *τ*^2^. Statistical heterogeneity was assessed using the *I*^*2*^ statistic and interpreted according to the guidelines in Chapter 9.5.2 of the Cochrane Handbook (Higgins 2011). This statistic expresses the percentage of variation across studies. Heterogeneity of *I*^*2*^ < 25% was considered low, between 25 and 75% was considered moderate, and > 75% was considered high. Subgroup analyses based on the geographic regions in which the studies were performed were conducted to detect sources of heterogeneity. To assess statistically significant differences between two or more subgroups, confidence intervals were compared. If the confidence intervals overlapped, then the differences were considered statistically insignificant [33].

## Results

### Study identification

**Fig. 1:**
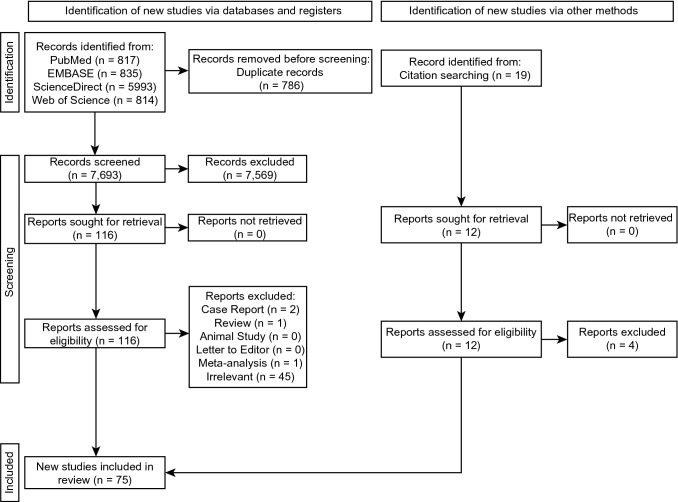
A flowchart depicting the study selection process according to PRISMA (Preferred Reporting Items for Systematic Reviews and Meta-Analysis) 2020 Guidelines

The study identification process is presented in Fig. 1. After extensive searching through the major databases (PubMed, Embase, ScienceDirect, Web of Science), 7,693 studies were initially identified. A further 19 were identified through citation searching; 124 studies were assessed by full text for potential eligibility, of which 49 were deemed ineligible. Thus, 75 studies were included in the meta-analysis.

### Characteristics of included studies

The characteristics of included studies are presented in Table 2. A total of 75 studies (*n* = 22,202 subjects) were considered eligible and were included in the meta-analysis. In total, there were 29 imaging studies and 46 cadaveric studies. The studies spanned the years 1982 to 2022 and originated from Africa, Asia, Europe, North America, and South America.

**Table 2:** Characteristics of studies included in the meta-analysis

Study	Country of origin	Type of investigation	Sample size by subjects (*n*)	Sample size by sides
Ajmani et al. [1] Indian	India	Cadaveric	86	172
Ajmani et al. [1] Nigerian	Nigeria	Cadaveric	34	68
Anjankar et al. [2]	India	Cadaveric	65	130
Aoun et al. [5]	Lebanon	Imaging	58	116
Aoun and Nasseh [3]	Lebanon	Imaging	79	158
Aoun et al. [4]	Lebanon	Imaging	74	148
Apinhasmit et al. [6]	Thailand	Cadaveric	55	110
Ashwini and Jaishree [7]	India	Cadaveric	100	200
Awad et al. [8]	Egypt	Imaging	200	400
Ayoub et al. [9]	United States	Imaging	50	100
Badshah et al. [10]	Pakistan	Cadaveric	85	170
Bahşi et al. [11]	Turkey	Imaging	150	300
Beetge et al. [12]	South Africa	Imaging	77	154
Cagimni et al. [16]	Turkey	Cadaveric	120	240
Campbell et al. [17]	United States	Imaging	50	100
Cheung et al. [18]	China	Cadaveric	30	60
Chopra et al. [19]	India	Cadaveric	100	200
Chrcanovic and Custódio [20]	Brazil	Cadaveric	80	160
Das et al. [22]	United States	Imaging	100	200
Dave et al. [23]	India	Cadaveric	100	200
D’Souza et al. [21]	India	Cadaveric	40	80
Douglas and Wormald [24]	Australia	Cadaveric	21	42
Duruel et al. [25]	United States	Imaging	131	262
Fonseka et al. [27]	Sri Lanka	Imaging	50	100
Fu et al. [28]	United States	Cadaveric	11	22
Gibelli et al. [29]	Italy	Cadaveric	100	200
Hassanali and Mwaniki [31]	Africa	Cadaveric	125	250
Howard-Swirzinski et al. [34]	United States	Imaging	500	1000
Hwang et al. [35]	South Korea	Imaging	50	100
Ikuta et al. [36]	Brazil	Imaging	50	100
Ilayperuma et al. [37]	Sri Lanka	Cadaveric	136	272
Jaffar and Hamadah [38]	Iraq	Cadaveric	50	100
Kaffe et al. [39]	Israel	Imaging	58	116
Kang et al. [40]	South Korea	Imaging	107	214
Kaur et al. [41]	India	Cadaveric	100	200
Klosek and Rungruang [42]	Thailand	Cadaveric	41	82
Kumar et al. [43]	India	Cadaveric	100	200
Lacerda-Santos et al. [44]	Brazil	Imaging	60	120
Langenegger et al. [45]	South Africa	Cadaveric	100	200
Lim et al. [47]	South Korea	Imaging	147	294
Lopes et al. [48]	Brazil	Cadaveric	94	188
Malamed and Trieger [49]	Mixed origin	Cadaveric	204	408
McKinney et al. [50]	United States	Imaging	10	20
Methathrathip et al. [52]	Thailand	Cadaveric	105	210
Narayan et al. [53]	India	Cadaveric	35	70
Nascimento et al. [54]	Brazil	Cadaveric	100	200
Nimigean et al. [55]	Romania	Cadaveric	100	200
Ortug and Uzel [56]	Turkey	Cadaveric	97	194
Piagkou et al. [57]	Greece	Cadaveric	71	142
Priya et al. [58]	India	Cadaveric	132	264
Rapado-González et al. [59]	Spain	Imaging	150	300
Rapado-González et al. [60]	Spain	Imaging	110	220
Renu [61]	India	Cadaveric	100	200
Reshmi [62]	India	Cadaveric	50	100
Safavi et al. [63]	Iran	Imaging	128	256
Salcedo et al. [64]	Chile	Cadaveric	31	62
Saralaya and Nayak [65]	India	Cadaveric	132	264
Sharma and Garud [66]	India	Cadaveric	100	200
Sheikhi et al. [67]	Iran	Imaging	138	276
Siddiqui et al. [68]	India	Cadaveric	98	196
Soto et al. [70]	Colombia	Cadaveric	50	100
Suzuki et al. [71]	Japan	Cadaveric	20	40
Teixeira et al. [73]	Brazil	Cadaveric	141	282
Thunyacharoen et al. [74]	Thailand	Cadaveric	200	400
Tomaszewska et al. [75]	Poland	Imaging	1200	2400
Tomaszewska et al. [77]	Poland	Imaging	1350	2700
Tomaszewska et al. [76]	Poland	Imaging	1500	3000
Urbano et al. [78]	Brazil	Cadaveric	43	86
Valizadeh et al. [79]	Iran	Imaging	148	296
Vidulasri and Thenmozhi [80]	India	Cadaveric	50	100
Vikraman et al. [81]	India	Cadaveric	30	60
Vinay et al. [82]	India	Cadaveric	150	300
Viveka and Kumar [83]	India	Imaging	44	88
Wang et al. [84]	China	Cadaveric	100	200
Westmoreland and Blanton [85]	India	Cadaveric	300	600
Wu et al. [86]	China	Imaging	120	240

Table displaying characteristics of the 75 studies that were included in the meta-analysis, sorted in alphabetical order (A–Z) by last names of the first authors of the studies. The study characteristics included country of study origin, whether the study subjects were either dry skulls (i.e., cadaveric studies) or CBCT (cone-beam computed tomography) scans (i.e., imaging studies), number of subjects in the studies, and the number of sides included in each study

### Quality assessment

Application of the AQUA tool criteria revealed that 41 studies (54.7%) in this meta-analysis had a “low” risk of bias while 34 studies (45.3%) had a “high” risk of bias in domain one (objective(s) and characteristics of the subject). In domain two (study design), 70 studies (93.3%) presented a “low” risk of bias and 5 studies (6.7%) a “high” risk. In contrast, 60 studies (80%) were assessed as having “low” risk of bias in domain three (methodology characterization) and 15 studies (20%) were assessed as having “high” risk of bias. In domain four (descriptive anatomy), 61 studies (81.3%) had a “low” risk of bias while the remaining 14 studies (18.7%) had a “high” risk of bias. Lastly, in domain five (reporting of results), 58 studies (77.3%) had a “low” risk of bias and 17 studies (22.7%) had a “high” risk of bias. Details of the risk of bias assessment using the AQUA tool criteria are shown in Table 3.

**Table 3:** Summary of results of the AQUA tool used to evaluate the risk of bias assessment

Study	Risk of bias				
	Objective(s) and study characteristics	Study design	Methodology characterization	Descriptive anatomy	Reporting of results
Ajmani et al. [1]	High	Low	Low	High	High
Anjankar et al. [2]	High	Low	Low	Low	Low
Aoun et al. [5]	Low	Low	Low	Low	Low
Aoun and Nasseh [3]	Low	Low	Low	Low	Low
Aoun et al. [4]	Low	Low	Low	Low	Low
Apinhasmit et al. [6]	Low	Low	Low	Low	High
Ashwini and Jaishree [7]	High	Low	Low	Low	Low
Awad et al. [8]	Low	Low	Low	Low	Low
Ayoub et al. [9]	Low	Low	Low	Low	Low
Badshah et al. [10]	High	Low	High	Low	Low
Bahşi et al. [11]	Low	Low	Low	Low	Low
Beetge et al. [12]	High	Low	Low	Low	Low
Cagimni et al. [16]	High	Low	Low	Low	High
Campbell et al. [17]	High	Low	High	Low	Low
Cheung et al. [18]	High	High	High	High	Low
Chopra et al. [19]	High	Low	Low	Low	High
Chrcanovic and Custódio [20]	High	Low	Low	Low	Low
Das et al. [22]	High	Low	High	Low	Low
Dave et al. [23]	High	Low	High	High	Low
D’Souza et al. [21]	Low	Low	Low	Low	Low
Douglas and Wormald [24]	High	Low	Low	Low	Low
Duruel et al. [25]	Low	Low	Low	Low	Low
Fonseka et al. [27]	Low	Low	Low	Low	Low
Fu et al. [28]	High	Low	Low	Low	High
Gibelli et al. [29]	Low	Low	Low	Low	Low
Hassanali and Mwaniki [31]	Low	Low	Low	Low	Low
Howard-Swirzinski et al. [34]	Low	Low	Low	Low	Low
Hwang et al. [35]	Low	Low	Low	Low	Low
Ikuta et al. [36]	Low	Low	Low	Low	Low
Ilayperuma et al. [37]	High	Low	Low	Low	Low
Jaffar and Hamadah [38]	High	Low	High	Low	Low
Kaffe et al. [39]	High	High	Low	Low	Low
Kang et al. [40]	High	Low	Low	Low	High
Kaur et al. [41]	Low	Low	Low	Low	Low
Klosek and Rungruang [42]	Low	Low	Low	Low	Low
Kumar et al. [43]	High	Low	Low	Low	Low
Lacerda-Santos, et al. [44]	Low	Low	Low	Low	Low
Langenegger et al. [45]	Low	Low	Low	Low	High
Lim et al. [47]	Low	Low	Low	Low	Low
Lopes et al. [48]	Low	Low	High	Low	Low
Malamed and Trieger [49]	High	High	High	High	High
McKinney et al. [50]	High	Low	Low	Low	High
Methathrathip et al. [52]	Low	Low	Low	High	High
Narayan et al. [53]	Low	Low	Low	Low	Low
Nascimento et al. [54]	Low	Low	Low	Low	Low
Nimigean et al. [55]	High	Low	Low	Low	Low
Ortug and Uzel [56]	Low	Low	Low	Low	Low
Piagkou et al. [57]	High	Low	Low	Low	High
Priya et al. [58]	High	Low	Low	Low	Low
Rapado-González et al. [59]	Low	Low	Low	Low	Low
Rapado-González et al. [60]	Low	Low	High	High	Low
Renu [61]	High	Low	Low	Low	Low
Reshmi [62]	High	High	High	High	High
Safavi et al. [63]	Low	Low	Low	Low	Low
Salcedo et al. [64]	Low	Low	Low	Low	High
Saralaya and Nayak [65]	High	Low	Low	Low	Low
Sharma and Garud [66]	High	Low	Low	Low	Low
Sheikhi et al. [67]	Low	Low	Low	High	Low
Siddiqui et al. [68]	High	Low	Low	High	High
Soto et al. [70]	High	High	High	High	Low
Suzuki et al. [71]	Low	Low	Low	Low	Low
Teixeira et al. [73]	Low	Low	Low	High	Low
Thunyacharoen et al. [74]	Low	Low	Low	Low	Low
Tomaszewska et al. [75]	Low	Low	Low	Low	Low
Tomaszewska et al. [77]	Low	Low	Low	Low	High
Tomaszewska et al. [76]	Low	Low	Low	Low	Low
Urbano et al. [78]	High	Low	High	High	High
Valizadeh et al. [79]	Low	Low	Low	Low	Low
Vidulasri and Thenmozhi [80]	High	Low	Low	Low	Low
Vikraman et al. [81]	High	Low	High	High	High
Vinay et al. [82]	High	Low	Low	Low	Low
Viveka and Kumar [83]	Low	Low	Low	Low	Low
Wang et al. [84]	Low	Low	High	Low	Low
Westmoreland and Blanton [85]	Low	Low	High	High	Low
Wu et al. [86]	Low	Low	Low	Low	Low

### Distance between the greater palatine foramen and selected anatomical landmarks

The results of the meta-analysis regarding the distance between the greater palatine foramen and surrounding anatomical landmarks are presented in Table 4. A total of 8 studies [8, 11, 29, 35, 56, 63, 77, 83] (*n* = 2358 subjects) reported data on the distance from the greater palatine foramen to the posterior nasal spine (GPF–PNS). The pooled mean, across the eight studies, was calculated to be 17.21 mm (95% CI = 16.34–18.09 mm). The *Q* test showed high heterogeneity (*Q* = 345.96; *p* < 0.0001), which was confirmed by the *I*^*2*^ test (98.0%; 95% CI = 97.2–98.6%). To explore the source of heterogeneity, the studies were subdivided into groups based on geographical location. For both subgroups, heterogeneity was still high: for Asian studies *Q* = 198.31 (*p* < 0.0001), *I*^*2*^ = 98.5% (95% CI = 97.6–99.0%) and for European studies *Q* = 105.84 (*p* < 0.0001), *I*^*2*^ = 99.1% (95% CI = 98.2–99.5%).

**Table 4:** Distance between the greater palatine foramen and surrounding anatomical landmarks

	Total number of studies	Total number of subjects	Pooled mean (95% CI) [mm]	Cochrane’s *Q*	*I*^*2*^ (95% CI) [%]	*p* value
GPF–PNS^a^	8	2358	17.21 (16.34–18.09)	345.96	98.0 (97.2–98.6)	*p* < 0.0001
Asia	4	380	17.06 (15.57–18.56)	198.31	98.5 (97.6–99.0)	*p* < 0.0001
Europe	2	1450	18.04 (15.94–20.15)	105.84	99.1 (98.2–99.5)	*p* < 0.0001
GPF–PBHP	24	4349	2.56 (1.90–3.22)	276,374.89	100.0	*p* = 0
Africa	2	465	3.71 (0.83–6.59)	43.02	97.7 (94.3–99.0)	*p* < 0.0001
Asia	17	2030	4.16 (3.17–5.15)	262,382.79	100.0	*p* = 0
Europe	3	1600	4.18 (1.83–6.54)	79.92	97.5 (95.1–98.7)	*p* < 0.0001
South America	2	254	3.41 (0.53–6.30)	0.11	0.0	*p* = 0.74
GPF–ANS^b^	4	365	46.24 (44.30–48.18)	90.68	96.7 (94.1–98.2)	*p* < 0.0001
GPF–MMS	38	5379	15.22 (15.00–15.43)	10,090.72	99.6 (99.6–99.7)	*p* = 0
Africa	3	565	15.13 (14.36–15.89)	42.13	95.3 (89.4–97.9)	*p* = 0.075
Asia	26	2506	15.14 (14.88–15.40)	9776.26	99.7 (99.7–99.8)	*p* = 0
Europe	4	1773	15.76 (15.10–16.42)	68.67	95.6 (91.7–97.7)	*p* < 0.0001
South America	5	535	15.21 (14.61–15.81)	56.23	92.9 (86.4–96.3)	*p* < 0.0001
GPF–IF	23	4404	37.32 (36.19–38.45)	3837.15	99.4 (99.4–99.5)	*p* = 0
Africa	2	520	38.23 (37.70–38.75)	2.68	62.6 (62.5–62.6)	*p* < 0.0001
Asia	16	2113	36.87 (35.51–38.22)	2078.70	99.3 (99.0–99.5)	*p* < 0.0001
Europe	2	1450	36.79 (31.69–41.89)	353.06	99.7 (99.6–99.8)	*p* < 0.0001
South America	3	321	39.47 (35.81–43.12)	173.63	98.8 (98.1–99.3)	*p* < 0.0001

*CI* confidence interval, *GPF* greater palatine foramen, *PNS* posterior nasal spine, *PBHP* posterior border of hard palate, *ANS* anterior nasal spine, *MMS* midline maxillary suture, *IF* incisive foramen

^a^Two studies [8, 63] were excluded from the subgroup analysis due to being the only studies in their own respective subgroups

^b^Subgroup analysis for GPF–ANS was not performed due to the low number of studies

A total of 24 studies [1, 7, 8, 10, 16, 19–21, 29, 37, 38, 41, 43, 48, 58, 59, 65, 66, 77, 80, 82, 84, 85] (*n* = 4349 subjects) reported data on the distance from the greater palatine foramen to the posterior border of the hard palate (GPF–PBHP). The pooled mean, across the 24 studies, was calculated to be 2.56 mm (95% CI = 1.90–3.22 mm). The *Q* test showed high heterogeneity (*Q* = 274,522.83; *p* < 0.0001), which was confirmed by the *I*^*2*^ test (100.0%). Subgroup analysis, based on geographical region, was performed to investigate heterogeneity. For South American studies, the *Q* test showed almost no heterogeneity (*Q* = 0.11; *p* = 0.74), confirmed by the *I*^*2*^ test (0.0%). For the other geographical regions, heterogeneity was still high: for Asian studies *Q* = 261,423.78 (*p* < 0.0001), *I*^*2*^ = 100.0%, and for European studies *Q* = 79.92 (*p* < 0.0001), *I*^*2*^ = 97.5% (95% CI = 95.1–98.7%).

A total of 4 studies [5, 27, 40, 59] (*n* = 365 subjects) reported data on the distance from the greater palatine foramen to the anterior nasal spine (GPF–ANS). The pooled mean, across the four studies, was calculated to be 46.24 mm (95% CI = 44.30–48.18 mm). The *Q* test showed high heterogeneity (*Q* = 90.68; *p* < 0.0001), which was confirmed by the *I*^*2*^ test (96.7%). Subgroup analysis based on geographical region was not performed due to the low number of studies; there were only two possible subgroups and one of these contained only one study, precluding the possibility of pooling the mean using meta-analysis.

A total of 38 studies [1, 5, 7, 8, 10, 11, 16, 19–22, 27, 29, 36–38, 40–45, 48, 56–59, 63, 65, 66, 73, 77, 80–85] (*n* = 5479 subjects) reported data on the distance from the greater palatine foramen to the median maxillary suture (GPF–MMS). The pooled mean, across the 38 studies, was calculated to be 15.22 mm (95% CI = 15.00–15.43 mm). The *Q* test showed high heterogeneity (*Q* = 10,090.72; *p* = 0), which was confirmed by the *I*^*2*^ test (99.6%; 95% CI = 99.6–99.7%). Subgroup analysis, based on geographical location of the studies, was performed to explore the source of this heterogeneity. For African studies, the *Q* test showed high heterogeneity (*Q* = 42.13; *p* = 0.075), confirmed by the *I*^*2*^ test (95.3%; 95% CI = 89.4–97.9%). For the other geographical regions, heterogeneity was significantly higher: for Asian studies *Q* = 9776.26 (*p* < 0.0001), *I*^*2*^ = 99.7% (95% CI = 99.7–99.8%), for European studies *Q* = 68.67 (*p* < 0.0001), *I*^*2*^ = 95.6% (95% CI = 91.7–97.7%), and for South American studies *Q* = 56.23 (*p* < 0.0001), *I*^*2*^ = 92.9% (95% CI = 86.4–96.3%).

A total of 23 studies [7, 8, 10, 11, 20, 29, 40, 41, 43, 44, 47, 54, 56, 58, 65, 66, 73, 74, 77, 80, 82, 83, 86] (*n* = 3164 subjects) reported data on the distance from the greater palatine foramen to the incisive fossa (GPF–IF). The pooled mean, across the 23 studies, was calculated to be 37.32 mm (95% CI = 36.19–38.45 mm). The *Q* test showed high heterogeneity (*Q* = 3837.15; *p* = 0), which was confirmed by the *I*^*2*^ test (99.4%; 95% CI = 99.4–99.5%). Subgroup analysis, based on geographic location of the studies, was performed to explore sources of heterogeneity. For African studies, the *Q* test showed high heterogeneity (*Q* = 2.68; *p* < 0.0001), confirmed by the *I*^*2*^ test (62.6%; 95% CI = 62.5–62.6%), for Asian studies *Q* = 2078.70 (*p* < 0.0001), *I*^*2*^ = 98.7% (95% CI = 99.0–99.5%), for European studies *Q* = 353.06 (*p* < 0.0001), *I*^*2*^ = 99.7% (95% CI = 99.6–99.8), and for South American studies *Q* = 173.63 (*p* < 0.0001), *I*^*2*^ = 98.8% (95% CI = 98.1–99.3%).

### Location of the greater palatine foramen in relation to maxillary molars

The results of the meta-analysis regarding the location of the GPF in relation to the maxillary molar teeth are presented in Table 5. Only two studies [19, 27] (*n* = 284 subjects) reported data on the prevalence of the greater palatine foramen being located “anterior to the 2nd maxillary molar teeth”. The pooled prevalence, across the two studies, was calculated to be 3.27% (95% CI = 0.45–20.29%). The statistical significance of the *Q* test (*Q* = 6.67, *df* = 1, *p* = 0.0098) allowed the null hypothesis of homogeneity to be rejected. The *I*^*2*^ test showed moderate to high heterogeneity (*I*^*2*^ = 85.0%; 95% CI = 58.5–94.6%).

**Table 5:** The location of the greater palatine foramen in relation to maxillary molars

	Total number of studies	Total number of subjects	Pooled prevalence (95% CI) [%]	Cochrane’s *Q*	*I*^*2*^ (95% CI) [%]	*p* value
Anterior to M2^a^	2	284	3.3 (0.5–20.3)	6.67	85.0 (58.5–94.6)	*p* = 0.0098
Opposite M2^b^	33	8852	5.0 (3.2–3.9)	371.78	91.4 (89.0–93.3)	*p* < 0.0001
Africa	4	980	6.2 (1.0–30.0)	10.86	72.4 (48.5–93.3)	*p* = 0.0045
Asia	23	4731	3.8 (2.2–6.3)	160.52	86.3 (80.9–90.7)	*p* < 0.0001
Europe	2	2800	16.0 (9.9–26.9)	3.68	72.8 (30.3–89.4)	*p* = 0.055
South America	2	162	4.3 (2.2–9.6)	1.05	5.0 (0.0–11.4)	*p* = 0.30
Between M2 and M3	37	9496	19.3 (15.3–24.0)	547.71	94.5 (93.2–95.6)	*p* < 0.0001
Africa	4	980	11.4 (2.9–35.6)	54.62	96.3 (92.5–98.2)	*p* < 0.0001
Asia	27	5366	20.6 (17.3–24.4)	155.95	86.5 (81.0–90.5)	*p* < 0.0001
Europe	3	2907	11.8 (5.9–22.1)	21.87	90.9 (77.5–96.3)	*p* < 0.0001
South America	2	222	18.1 (2.1–69.6)	32.83	97.0 (92.9–98.7)	*p* < 0.0001
Opposite M3	38	9754	64.9 (58.7–70.7)	610.61	94.8 (93.5–95.8)	*p* < 0.0001
Africa	4	980	49.5 (46.4–52.6)	76.94	97.4 (95.0–98.6)	*p* < 0.0001
Asia	27	5366	66.7 (65.4–67.9)	363.88	94.0 (92.4–95.6)	*p* < 0.0001
Europe	3	2907	74.7 (73.1–76.2)	0.21	0.0	*p* = 0.90
South America	3	322	65.8 (60.5–70.8)	33.96	94.1 (86.7–97.4)	*p* < 0.0001
Distal to M3	31	8608	6.0 (3.7–9.6)	1183.69	96.7 (96.1–97.3)	*p* < 0.0001
Asia	24	4841	7.7 (7.0– 8.6)	263.34	89.0 (84.5–92.2)	*p* < 0.0001
Europe	3	2907	2.4 (1.9–3.0)	10.93	81.7 (49.2–93.4)	*p* = 0.0042
South America	2	260	25.8 (20.8–31.4)	26.11	96.2 (90.7–98.4)	*p* < 0.0001

*CI* confidence interval, *M2* second maxillary molar, *M3* third maxillary molar

^a^Subgroup analysis was not performed due to the low number of studies

^b^Two studies [28, 49] were excluded from the subgroup analysis because they were the only studies in their own respective subgroups

A total of 33 studies [1, 2, 5, 7, 8, 10, 11, 19, 21, 22, 27, 28, 31, 36–39, 41, 43, 45, 49, 52, 55, 58, 61, 63–65, 77, 79, 81, 82, 85] (*n* = 8,852 subjects) reported data on the prevalence of the greater palatine foramen being located “opposite the 2nd maxillary molar teeth”. The pooled prevalence, across the 33 studies, was calculated to be 5.0% (95% CI = 3.2–3.9%). The statistical significance of the *Q* test (*Q* = 371.78, *p* < 0.0001) allowed the null hypothesis of homogeneity to be rejected. The *I*^*2*^ test showed high heterogeneity (*I*^*2*^ = 91.4%; 95% CI = 89.0–93.3%).

A total of 37 studies [1, 2, 5, 7, 8, 10, 11, 19–22, 27, 28, 31, 37–39, 41, 43, 45, 52, 53, 55, 57, 58, 61, 63–65, 68, 74, 77, 79, 81, 82, 85] (*n* = 9,496 subjects) reported data on the greater palatine foramen being located “between the 2nd and 3rd maxillary molar teeth”. The pooled prevalence, across the 37 studies, was calculated to be 19.3% (95% CI = 15.3–24.0%). The statistical significance of the *Q* test (*Q* = 547.61, *p* < 0.0001) allowed the null hypothesis of homogeneity to be rejected. The *I*^*2*^ test showed high heterogeneity (*I*^*2*^ = 94.5%; 95% CI = 93.2–95.6%).

**Fig. 2:**
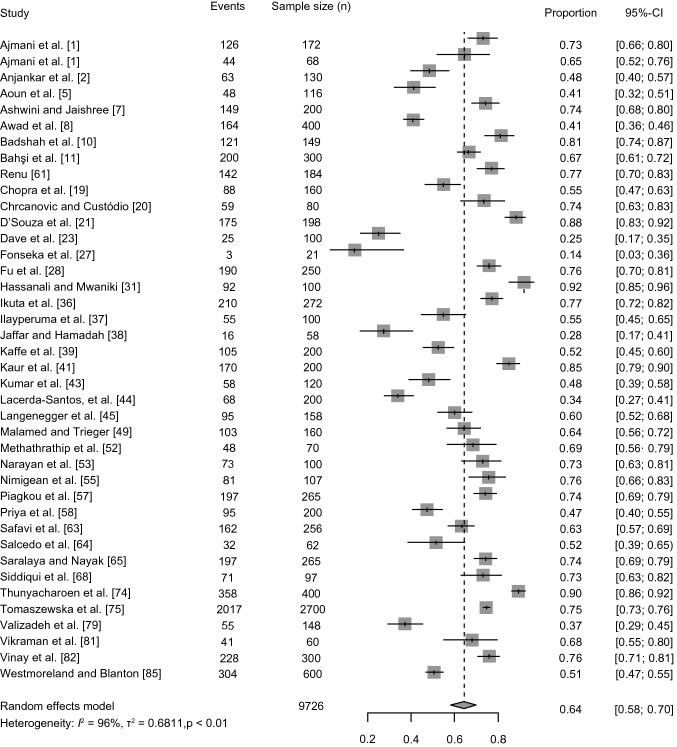
Forest plot depicting the prevalence of the greater palatine foramen positioned opposite the third maxillary molar teeth. Studies were sorted in order of the reported proportion, defined as the ratio of the number of events (GPF being located opposite M3) to the total number of subjects in the study

A total of 38 studies [1, 2, 5, 7, 8, 10, 11, 19–22, 27, 28, 31, 36–39, 41, 43, 45, 49, 52, 53, 55, 57, 58, 61, 63–65, 68, 74, 77, 79, 81, 82, 85] (*n* = 9754 subjects) reported data on the greater palatine foramen being located “opposite the 3rd maxillary molar teeth”. The pooled prevalence, across the 38 studies, was calculated to be 64.9% (95% CI = 58.7–70.7%). The statistical significance of the *Q* test (*Q* = 610.61, *p* < 0.0001) allowed the null hypothesis of homogeneity to be rejected. The *I*^*2*^ test showed high heterogeneity (*I*^*2*^ = 94.8%; 95% CI = 93.5–95.8%). The results of the meta-analysis are shown as a forest plot in Fig. 2.

A total of 31 studies [1, 2, 5, 7, 8, 11, 19–21, 27, 36–39, 41, 43, 45, 52, 53, 55, 57, 58, 61, 63, 65, 68, 74, 77, 81, 82, 85] (*n* = 7,282 subjects) reported data on the greater palatine foramen (GPF) being located “distal to the 3rd maxillary molar”. The pooled prevalence, across the 31 studies, was calculated to be 6.0% (95% CI = 3.7–9.6%). The statistical significance of the *Q* test (*Q* = 1183.6, *p* < 0.0001) allowed the null hypothesis of homogeneity to be rejected. The *I*^*2*^ test showed high heterogeneity (*I*^*2*^ = 96.7%; 95% CI = 96.1–97.3%).

### Morphometric parameters of the greater palatine foramen

The results of the meta-analysis regarding the morphometric parameters of the GPF are presented in Table 6. A total of 13 studies [4, 5, 8, 11, 12, 25, 27, 35, 41, 57, 59, 66, 77] (*n* = 3,066 subjects) reported data on the anteroposterior (AP) diameter of the greater palatine foramen. The pooled mean, across the 11 studies, was calculated to be 5.34 mm (95% CI = 4.99–5.68 mm). The statistical significance of the *Q* test (*Q* = 491.85, *df* = 10, *p* < 0.0001) allowed the null hypothesis of homogeneity to be rejected. The *I*^*2*^ test showed high heterogeneity (*I*^*2*^ = 98.0%; 95% CI = 97.3–98.5%).

**Table 6:** Size and shape of the greater palatine foramen

	Total number of studies	Total number of subjects	Pooled estimate (95% CI)	Cochrane’s *Q*	*I*^*2*^ (95% CI) [%]	*p* value
AP diameter^a^ (mm)	13	3066	5.34 (4.99–5.68)	491.85	98.0 (97.3–98.5)	*p* < 0.0001
Africa	2	477	4.64 (3.88–5.40)	76.48	98.7 (97.2–99.4)	*p* < 0.0001
Asia	6	537	5.29 (4.84–5.74)	390.81	98.7 (98.2–99.1)	*p* < 0.0001
Europe	3	1721	5.50 (4.88–6.13)	91.48	97.8 (95.9–98.8)	*p* < 0.0001
LM diameter (mm)	14	4803	2.77 (2.58–2.96)	967.40	98.2 (98.3–98.9)	*p* < 0.0001
Africa	2	677	2.51 (2.12–2.90)	62.59	96.8 (93.5–98.4)	*p* = 0.0002
Asia	5	855	2.65 (2.34–2.96)	341.01	98.5 (97.9–99.0)	*p* < 0.0001
Europe	5	3271	3.04 (2.74–3.35)	105.66	96.2 (93.5–97.8)	*p* < 0.0001
Oval/ovoid shape^b^ (%)	6	986	77.8 (57.6–90.0)	150.22	96.7 (94.7–97.9)	*p* < 0.0001
Asia	3	516	93.2 (74.1–98.5)	35.28	94.3 (87.3–97.5)	*p* < 0.0001
South America	2	250	52.2 (40.9–63.2)	2.57	61.1 (12.0–82.8)	*p* = 0.11
Round shape^c^ (%)	4	670	9.4 (3.3–23.8)	44.43	91.0 (82.3–95.4)	*p* < 0.0001
Slit/lancet shape^c^ (%)	4	676	8.4 (2.4–25.8)	56.20	92.9 (86.6–96.2)	*p* < 0.0001
Other shape^c^ (%)	2	330	35.3 (14.3–64.0)	20.78	95.2 (87.9–98.1)	*p* < 0.0001

*CI* confidence interval, *AP* anteroposterior, *LM* lateromedial

^a^Two studies [12, 25] were excluded from the subgroup analysis because they were the only studies in their own respective subgroups

^b^One study [60] was excluded from the subgroup analysis

^c^Meta-analysis for the greater palatine foramen shapes “Round”, “Slit/lancet”, and “Other” were not followed up by subgroup analysis due to the low number of studies

A total of 14 studies [6, 8, 10–12, 35, 41, 45, 52, 55, 57, 59, 76, 77] (*n* = 4,803 subjects) reported data on the lateromedial (LM) diameter of the greater palatine foramen. The pooled mean, across the 12 studies, was calculated to be 2.77 mm (95% CI = 2.58–2.96 mm). The statistical significance of the *Q* test (*Q* = 967.40, *p* < 0.0001) allowed the null hypothesis of homogeneity to be rejected. The *I*^*2*^ test showed high heterogeneity (*I*^*2*^ = 98.2%; 95% CI = 98.3–98.9%).

### Morphology of the greater palatine foramen

The results of the meta-analysis regarding the morphology of the GPF are presented in Table 6. A total of 6 studies [6–8, 19, 41, 48, 60, 64] (*n* = 986 subjects) reported data on the prevalence of the greater palatine foramen being “oval/ovoid” in shape. The pooled prevalence, across the six studies, was calculated to be 77.8% (95%CI = 57.6–90.0%). The statistical significance of the *Q* test (*Q* = 150.22, *p* < 0.0001) allowed the null hypothesis of homogeneity to be rejected. The *I*^*2*^ test showed high heterogeneity (*I*^*2*^ = 96.7%; 95% CI = 94.7–97.9%).

A total of 4 studies [7, 8, 41, 48, 60, 64] (*n* = 670 subjects) reported data on the prevalence of the greater palatine foramen being “round” in shape. The pooled prevalence, across the four studies, was calculated to be 9.4% (95% CI = 3.3–23.8%). The statistical significance of the *Q* test (*Q* = 44.43, *p* < 0.0001) allowed the null hypothesis of homogeneity to be rejected. The *I*^*2*^ test showed moderate to high heterogeneity (*I*^*2*^ = 91.0%; 95% CI = 82.3–95.4%).

A total of 4 studies [19, 41, 48, 60, 64] (*n* = 676 subjects) reported data on the prevalence of the greater palatine foramen being “slit/lancet” in shape. The pooled prevalence, across the four studies, was calculated to be 8.4% (95% CI = 2.4–25.8%). The statistical significance of the *Q* test (*Q* = 56.20, *p* < 0.0001) allowed the null hypothesis of homogeneity to be rejected. The *I*^*2*^ test showed moderate to high heterogeneity (*I*^*2*^ = 92.9%; 95% CI = 86.6–96.2%).

Only 2 studies [6, 41, 60] (*n* = 330 subjects) reported data on the prevalence of the greater palatine foramen being “other” in shape. The pooled prevalence, across the two studies, was calculated to be 35.3% (95% CI = 14.3–64.0%). The statistical significance of the *Q* test (*Q* = 20.78, *p* < 0.0001) allowed the null hypothesis of homogeneity to be rejected. The *I*^*2*^ test showed high heterogeneity (*I*^*2*^ = 95.2%; 95% CI = 87.9–98.1%).

### Direction of opening of the greater palatine foramen

The results of the meta-analysis regarding the direction of opening of the GPF into the oral cavity are presented in Table 7. A total of 10 studies [1, 2, 7, 37, 43, 65, 66, 68, 77, 82] (*n* = 4,534 subjects) reported data on the prevalence of the greater palatine foramen opening into the oral cavity in the inferior–anterior–lateral direction. The pooled prevalence, across the ten studies, was calculated to be 14.41% (95% CI = 4.91–35.43%). The statistical significance of the *Q* test (*Q* = 873.78, *p* < 0.0001) allowed the null hypothesis of homogeneity to be rejected. The *I*^*2*^ test showed high heterogeneity (*I*^*2*^ = 99.0%; 95% CI = 98.7–99.2%).

**Table 7:** Direction of the opening of the greater palatine foramen into the oral cavity

	Total number of studies	Total number of subjects	Pooled prevalence (95% CI) [%]	Cochrane’s *Q*	*I*^*2*^ (95% CI) [%]	*p* value
I–A–L^a^	10	4534	14.41 (4.91–35.43)	873.78	99.0 (98.7–99.2)	*p* < 0.0001
Anterior	15	5864	30.11 (17.67–46.37)	1063.43	98.7 (98.4–98.9)	*p* < 0.0001
Asia	12	2804	32.74 (20.40–48.05)	453.05	97.6 (96.8–98.2)	*p* < 0.0001
Europe	2	2900	9.58 (5.45–16.31)	7.87	87.3 (64.8–95.4)	*p* = 0.0050
I–A–M	14	5312	54.54 (40.53–67.87)	886.74	98.4 (98.0–98.7)	*p* < 0.0001
Asia	10	1872	48.88 (36.66–61.24)	216.68	95.8 (94.0–97.1)	*p* < 0.0001
Europe	2	2900	82.55 (81.13–83.89)	0.05	0.0	*p* = 0.83
Vertical	13	5490	15.95 (5.78–37.00)	1406.94	99.1 (99.0–99.3)	*p* < 0.0001
Asia	9	2180	19.62 (5.25–51.84)	741.85	98.9 (98.6–99.2)	*p* < 0.0001
Europe	2	2900	5.17 (4.42–6.04)	0.01	0.0	*p* = 0.91

*CI* confidence interval, *I–A–L* inferior–anterior–lateral, *I–A–M*, inferior–anterior–medial

^a^Subgroup analysis for I–A–L was not performed due to the low number of studies; there were only two possible subgroups and one of these contained only one study, precluding the possibility of pooling the prevalence using meta-analysis

A total of 15 studies [1, 7, 20, 21, 37, 38, 41, 43, 55, 65, 66, 68, 77, 82, 84, 85] (*n* = 5,864 subjects) reported data on the prevalence of the greater palatine foramen opening anteriorly into the oral cavity. The pooled prevalence, across the 15 studies, was calculated to be 30.11% (95% CI = 17.67–46.37%). The statistical significance of the *Q* test (*Q* = 1063.43, *p* < 0.0001) allowed the null hypothesis of homogeneity to be rejected. The *I*^*2*^ test showed high heterogeneity (*I*^*2*^ = 98.7%; 95% CI = 98.4–98.9%).

A total of 15 studies [1, 2, 7, 20, 31, 37, 38, 41, 43, 55, 65, 66, 68, 77, 82] (*n* = 5,312 subjects) reported data on the prevalence of the greater palatine foramen opening in the inferior–anterior–medial direction into the oral cavity. The pooled prevalence, across the 15 studies, was calculated to be 54.54% (95% CI = 40.53–67.87%). The statistical significance of the *Q* test (*Q* = 886.74, *p* < 0.0001) allowed the null hypothesis of homogeneity to be rejected. The *I*^*2*^ test showed high heterogeneity (*I*^*2*^ = 98.4%; 95% CI = 98.0–98.7%).

A total of 13 studies [1, 20, 21, 31, 38, 43, 52, 55, 66, 77, 82, 84, 85] (*n* = 5,490 subjects) reported data on the prevalence of the greater palatine foramen opening in the vertical direction into the oral cavity. The pooled prevalence, across the 13 studies, was calculated to be 15.94% (95% CI = 5.78–37.00%). The statistical significance of the *Q* test (*Q* = 1406.94, *p* < 0.0001) allowed the null hypothesis of homogeneity to be rejected. The *I*^*2*^ test showed high heterogeneity (*I*^*2*^ = 99.2%; 95% CI = 99.0–99.3%).

### Other characteristics of the greater palatine canal

The results of the meta-analysis regarding other characteristics of the GPC are presented in Table 8. A total of 13 studies [3, 4, 23, 25, 34, 35, 50, 52, 59, 67, 70, 75, 76] (*n* = 4,798 subjects) reported data on the length of the greater palatine canal. The pooled mean, across the 13 studies, was calculated to be 26.97 mm (95% CI = 23.65–30.29 mm). The statistical significance of the *Q* test (*Q* = 17,900.35, *df* = 12, *p* < 0.0001) allowed the null hypothesis of homogeneity to be rejected. The *I*^*2*^ test showed high heterogeneity (*I*^*2*^ = 99.93%; 95% CI = 99.93–99.94%).

**Table 8:** Characteristics of the greater palatine canal

	Total number of studies	Total number of subjects	Pooled estimate (95% CI)	Cochrane’s *Q*	*I*^*2*^ (95% CI) [%]	*p* value
Length (mm)	13	4798	26.97 (23.65–30.29)	17,900.35	100.0	*p* = 0
Asia	5	597	28.19 (19.58–36.80)	6434.65	100.0	*p* = 0
Europe	3	2850	24.86 (16.01–33.71)	10,945.60	100.0	*p* = 0
North America	4	1251	25.78 (23.44–28.13)	280.19	98.9 (98.4–99.3)	*p* < 0.0001
AP diameter^a^ (mm)	3	360	4.61 (2.74–6.47)	486.64	99.6 (99.4–99.7)	*p* < 0.0001
Angle between the vertical plane and the axis of the GPC (°)	4	710	19.09 (9.20–28.99)	1297.44	99.7 (99.6–99.8)	*p* < 0.0001
Asia	2	510	10.76 (2.80–18.72)	225.37	99.6 (99.3–99.7)	*p* < 0.0001
North America	2	200	27.45 (18.74–36.16)	91.84	98.9 (97.9–99.4)	*p* < 0.0001
Angle between the transverse plane and the axis of the GPC* (°)	2	310	62.63 (53.32–71.94)	141.84	99.3 (98.7–99.6)	*p* < 0.0001

*CI* confidence interval, *AP* anteroposterior, *GPC* greater palatine canal

^a^Subgroup analyses were not performed due to the low number of studies; for each parameter, there were only two possible subgroups and one of these contained only one study, precluding the possibility of pooling the prevalence using meta-analysis

A total of 3 studies [3, 25, 59] (*n* = 360) reported data on the anteroposterior diameter of the upper opening of the greater palatine canal. The pooled mean, across the three studies, was calculated to be 3.88 mm (95% CI = 3.77–3.99 mm). The statistical significance of the *Q* test (*Q* = 486.64, *df* = 2, *p* < 0.0001) allowed the null hypothesis of homogeneity to be rejected. The *I*^*2*^ test showed high heterogeneity (*I*^*2*^ = 99.59%; 95% CI = 99.41–99.71%).

A total of 5 studies [9, 11, 17, 44, 52] (*n* = 710 subjects) reported data on the angle between the vertical plane and the axis of the greater palatine canal. The pooled mean, across the four studies, was calculated to be 19.09° (95% CI = 9.20–28.99°). The statistical significance of the *Q* test (*Q* = 1297.44, *df* = 4, *p* < 0.0001) allowed the null hypothesis of homogeneity to be rejected. The *I*^*2*^ test showed high heterogeneity (*I*^*2*^ = 99.69%; 95% CI = 99.61–99.75%).

A total of 2 studies [35, 52] (*n* = 310 subjects) reported data on the measured angle between the transverse plane and the axis of the greater palatine canal. The pooled mean, across the two studies, was calculated to be 62.63° (95% CI = 53.32–71.94°). The statistical significance of the *Q* test (*Q* = 141.84, *df* = 1, *p* < 0.0001) allowed the null hypothesis of homogeneity to be rejected. The *I*^*2*^ test showed high heterogeneity (*I*^*2*^ = 99.29%; 95% CI = 98.75–99.60%).

## Discussion

To date, the leading anesthesiology and surgery textbooks have offered only general descriptions regarding clinical localization of the greater palatine foramen (GPF) and greater palatine canal (GPC), often leading to inconsistencies in physician training [82]. Though a large number of studies have been conducted concerning the location and morphometric characteristics of the GPF and GPC, many of these publications report an ongoing difficulty in localizing these structures, and therefore identifying the GPA in clinical settings [30].

Locating the GPF in relation to maxillary molar teeth remains a fast and effective way for clinicians to estimate the location of the GPF. Our findings were consistent with those of a similar review by Tomaszewska et al. [75], which also revealed that the GPF is most commonly located opposite the third maxillary molar (M3). Our results add substantial value to the findings of Tomaszewska et al. [75]. The analysis of the prior review contained only 23 studies (*n* = 6927 subjects) and the pooled prevalence was estimated to be 63.9% with a 95% confidence interval ranging from 56.5 to 70.9%. Our review, which contained a total of 38 studies (*n* = 9,754 subjects) and a pooled prevalence of 64.9%, with a 95% confidence interval from 58.7 to 70.7% strengthens the validity of the findings of Tomaszewska et al. [75–77] with the addition of 15 studies, adding significantly to the overall sample size, and narrowing the 95% confidence interval.

An additional aspect to consider when referencing the GPF to the maxillary molars is the size and shape of the GPF. Our meta-analysis revealed that the GPF has an anteroposterior (AP) diameter of 5.34 mm and lateromedial (LM) diameter of 2.82 mm, representing the major and minor axes, respectively. This is consistent with our other findings that the GPF was described as “oval or ovoid” in shape in 77.78% of the population. A possible explanation for such AP elongation of the GPF, is that the AP dimension of the palate increases with the eruption of the posterior teeth.

In edentulous patients, the location of the GPF can be accurately triangulated using measured distances to easily identifiable landmarks, the most reliable of which are the median maxillary suture (MMS), the posterior border of the hard palate (PBHP), and the incisive foramen (IF), rather than the posterior nasal septum (PNS) and the anterior nasal septum (ANS). The topography of the hard palate with reference to the anatomical landmarks is of clinical importance also when obtaining free gingival and connective tissue grafts [42], where the distance from the GPF to the incisive foramen (GPF–IF) is used to estimate the possible length of the graft [20, 42, 64].

Furthermore, using GPF–IF and GPF–MMS, it is possible to derive the angle between the MMS and the line from the IF to the GPF, which Tomaszewska et al. [75] called the MMS–IF–GPF angle. Utilizing our findings for GPF–IF and GPF–MMS, we found the MMS–IF–GPF angle to be 24.07 degrees, which is consistent with the angle calculated by Saralaya and Nayak (21.1 and 21.2 degrees) [64] and Chrcanovic and Custódio (22.12 and 23.30 degrees) [20]. Knowing the MMS–IF–GPF angle may also be useful in determining the angle to be made by the needle for anesthetic infiltration into the GPF [20, 64].

In the setting of maxillary nerve block and hemostasis using the GPC approach, the length of the GPC is particularly relevant. For anesthesia, the needle must advance 30 mm, while for hemostasis, specifically during sinus surgery, it is recommended to infiltrate the needle as deep as 25 mm [75].

Our meta-analysis results suggest that anatomical variation of the direction of opening of the GPF may occur more frequently than previously thought. An inferior–anterior–medial (I–A–M) opening relative to the sagittal plane was found 54.54% of the time, considerably less than that was previously estimated at 82.1% [75]. The second most common direction of opening was in the anterior direction, occurring 30.11% of the time in our study, in stark contrast to 7.6% in the 2015 study by Tomaszewska et al. [77] The most common method of administering anesthesia via the GPF was to bend the needle to an angle of 30–45 degrees. In light of our findings, it may be advisable to administer anesthesia to the maxillary nerve by bending the needle to an angle closer to 30 degrees, as the smaller angle would mitigate the risk of puncturing the hard palate soft tissue in the case that the GPF opens in the anterior direction.

One notable variation in GPF anatomy, as shown in ultrasonographic imaging studies [22], is a bony ledge that partially covers the opening of the foramen; in the presence of this variation, the data collected and pooled on the direction of opening becomes a clinically difficult statistic, and represents another challenge the clinician must be aware of when inserting a needle into the GPF.

We met with several limitations during our systematic review which was the lack of studies which directly described the anatomy of the GPA; this prevents us from making conclusions about the course of the artery itself, at least distal to the GPF. Another issue was the heterogeneity of the included studies, both in terms of the parameters measured and the modalities used to measure them (e.g., imaging versus cadaveric studies). For instance, as mentioned above, different studies used different categories to report the location of the GPF in relation to the maxillary molars, as well as its shape (see also Tables 3, 4). On the other hand, some parameters—such as the distance between the GPF and the nasal spines—were only reported in a small number of studies (Table 2). The main limitation of the meta-analysis was the substantial heterogeneity among the included studies, which persisted even after subgroup analysis based on geographical region. The included studies featured little information on individual patient characteristics, such as gender, precluding a more detailed subgroup analysis. The majority of the studies were performed on dry adult skulls, and consequently, the majority of these studies also did not report gender or age, which posed a limitation when probing for possible sources of heterogeneity.

**Fig. 3:**
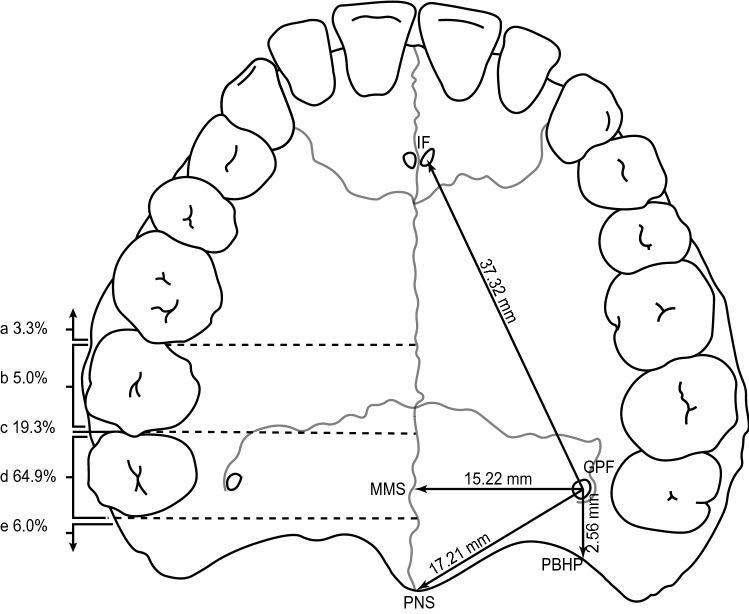
Illustration of the hard palate, displaying the greater palatine foramen in relation to anatomical landmarks and the maxillary molar teeth. The pooled mean distances from the greater palatine foramen (GPF) to four major anatomical landmarks (IF, MMS, PBHP, PNS) are shown on the right side of the diagram, while the pooled prevalence of the greater palatine foramen location in relation to the maxillary molar teeth are shown on the left side, (**a**–**e**). *GPF* greater palatine foramen, *IF* incisive foramen, *MMS* midline maxillary suture, *PBHP* posterior border of hard palate, *PNS* posterior nasal spine; **a** anterior to the mesial surface of the second maxillary molar; **b** opposite to the second maxillary molar; **c** between the second and the third maxillary molar; **d** opposite to the third maxillary molar; **e** distal to the third maxillary molar

We propose that the maxillary molar teeth, midline maxillary suture, posterior border of the hard palate, and the incisive foramen are the most reliable anatomical landmarks to accurately locate the GPF. Clinicians may expect to locate the foramen 15.00–15.44 mm from the midline maxillary suture, 1.90–3.22 mm from the posterior border of the hard palate, and 36.19–38.45 mm from the incisive foramen. The main findings are summarized in Fig. 3.

## Data Availability

All of the data that were extracted from the included studies during the data collection process and used to perform our analyses are stored in a repository and publicly available on Open Science Framework (https://osf.io/64thm/?view_only=d472458ec3084d9da9efefdfd396b605).
